# 4-Phenylbutyric Acid Reduces Endoplasmic Reticulum Stress in Chondrocytes That Is Caused by Loss of the Protein Disulfide Isomerase ERp57

**DOI:** 10.1155/2019/6404035

**Published:** 2019-10-29

**Authors:** Yvonne Rellmann, Isabel Gronau, Uwe Hansen, Rita Dreier

**Affiliations:** ^1^Institute of Physiological Chemistry and Pathobiochemistry, Muenster, Germany; ^2^Department of Life Sciences and Chemistry, Jacobs University Bremen, Bremen, Germany; ^3^Institute for Musculoskeletal Medicine, Muenster, Germany

## Abstract

**Objective:**

The integrity of cartilage depends on the correct synthesis of extracellular matrix (ECM) components. In case of insufficient folding of proteins in the endoplasmic reticulum (ER) of chondrocytes, ECM proteins aggregate, ER stress evolves, and the unfolded protein response (UPR) is initiated. By this mechanism, chondrocytes relieve the stress condition or initiate cell death by apoptosis. Especially persistent ER stress has emerged as a pathogenic mechanism in cartilage diseases, such as chondrodysplasias and osteoarthritis. As pharmacological intervention is not available yet, it is of great interest to understand cartilage ER stress in detail and to develop therapeutics to intervene.

**Methods:**

ERp57-deficient chondrocytes were generated by CRISPR/Cas9-induced KO. ER stress and autophagy were studied on mRNA and protein level as well as by transmission electron microscopy (TEM) in chondrocyte micromass or cartilage explant cultures of ERp57 KO mice. Thapsigargin (Tg), an inhibitor of the ER-residing Ca^2+^-ATPase, and 4-Phenylbutyric acid (4-PBA), a small molecular chemical chaperone, were applied to induce or inhibit ER stress.

**Results:**

Our data reveal that the loss of the protein disulfide isomerase ERp57 is sufficient to induce ER stress in chondrocytes. 4-PBA efficiently diffuses into cartilage explant cultures and diminishes excessive ER stress in chondrocytes dose dependently, no matter if it is induced by ERp57 KO or stimulation with Tg.

**Conclusion:**

ER-stress-related diseases have different sources; therefore, various targets for therapeutic treatment exist. In the future, 4-PBA may be used alone or in combination with other drugs for the treatment of ER-stress-related skeletal disorders in patients.

## 1. Introduction

During endochondral ossification, chondrocytes produce large amounts of extracellular matrix (ECM) components [1]. Prior to secretion, ECM proteins need to undergo posttranslational modification and folding in the endoplasmic reticulum (ER). Accordingly, the ER lumen contains resident folding complexes containing heat shock proteins, lectins, oxidoreductases, protein disulfide isomerases (PDIs), and peptidyl-prolyl *cis*/*trans* isomerases [2–4]. ECM protein folding often begins with glycosylation and subsequent trimming of the N-linked glycans. These processes enhance the solubility and allow the interaction with calnexin and calreticulin to promote folding [5, 6]. Both lectins also bind ERp57, a PDI which is in the focus of this study. ERp57 is responsible for a correct disulfide bridge formation in glycoproteins with unstructured disulfide-rich domains [7–10] and is part of the so-called calnexin/calreticulin cycle, a series of events which are repeated until the native conformation of a protein is finally achieved [6, 11]. Correctly folded proteins then move to the Golgi apparatus where posttranslational modification is completed and sorting into vesicles for the secretion into the ECM is established.

Different cellular conditions, e.g., phases of extraordinary protein demand, low oxygen tension, limited nutrient conditions, or mutations in ECM proteins can prevent proper protein folding and subsequent secretion [1]. The resulting accumulation of misfolded proteins in the ER is called ER stress and induces the unfolded protein response (UPR) [4]. In mammals, this quality control system is activated by three ER transmembrane stress sensors: Protein Kinase RNA-like Endoplasmic Reticulum Kinase (PERK), inositol requiring enzyme 1 (IRE1), and activating transcription factor 6 (ATF6). Under normal conditions, these sensors are inactive due to the binding of Binding immunoglobulin Protein (BiP). If unfolded proteins appear, BiP dissociates and binds to the stretched proteins. This activates signaling pathways initiating a general stop of cellular protein synthesis, an increased production of additional chaperones, and an advanced degradation of aggregated proteins by ER-associated degradation or autophagy. However, if the normal ER function cannot be relieved and ER stress prolongs, apoptosis is initiated via the transcription factor C/EBP homologous protein (Chop) [12].

Persistent ER stress is thought to be a pathogenic mechanism behind a variety of diseases, among them short-stature-diseases like metaphyseal chondrodysplasia type Schmid (MCDS), multiple epiphyseal dysplasia (MED), and pseudoachondrodysplasia (PSACH). Accordingly, various mouse models were used to study ER stress in skeletal disorders [13, 14]. In this context, we generated cartilage-specific ERp57 KO mice showing a chondrodysplasia-like phenotype which was pronounced during the pubertal growth spurt [15]. We detected growth plate chondrocytes with dilated ER structures, reduced proliferation, and enhanced apoptosis throughout the epiphyseal plates. ER stress was confirmed by the detection of higher BiP and Chop levels in ERp57 KO chondrocytes compared to WT cells. All existing data concerning chondrodysplasias substantiate that ER stress itself acts as a pathogenic factor [13, 14].

However, ER stress does not only affect skeletal development. Various studies describe a link between osteoarthritis (OA) and ER stress [1, 16–18]. It is known that elderly patients have a reduced ability to manage ER stress in the articular cartilage as the expression of ER chaperones and UPR proteins declines with age [19]. Accordingly, increased levels of apoptotic chondrocytes, a hallmark of advanced OA, occur [20].

Therapeutics targeting ER stress are under investigation for some time as ER stress is crucially involved in numerous other diseases such as metabolic disorders, neurodegeneration, and cancer [21]. Some compounds have already demonstrated therapeutic efficacy in animal and human studies. The present study focuses on the low molecular weight chemical chaperone 4-PBA. 4-PBA is approved for the treatment of urea cycle disorders, but the majority of investigations suggest that it acts as a chemical chaperone that attenuates ER stress in different cell types [22]. 4-PBA supports folding processes by interacting with hydrophobic regions in unfolded proteins, thus preventing their aggregation. Therefore, we tested the protective effect of 4-PBA in chondrocytes with ER stress induced by the loss of ERp57 or by Thapsigargin.

## 2. Materials and Methods

### 2.1. ERp57 KO Mice

Cartilage-specific ERp57 knockout (cKO) mice were generated as described [15]. C57BL76 wild-type (WT) and cKO mice were littermates derived from the breeding of homozygous ERp57 floxed, cre-positive, and cre-negative mice. The animals were maintained under specific pathogen-free conditions with food and water ad libitum and a 12-hour light/dark cycle in compliance with the German federal law for animal protection under the control of the North Rhine-Westphalia State Agency for Nature, Environment, and Consumer Protection (LANUV, NRW).

### 2.2. Cartilage Explant Cultures

Knee joint and femoral head cartilage was isolated from newborn cKO or WT mice, washed in phosphate-buffered saline (PBS), and transferred into Dulbecco's modified Eagle's medium (DMEM, Biochrom, Berlin, Germany) containing 60 *μ*g/ml *β*-aminopropionitrile fumarate, 25 *μ*g/ml sodium ascorbate, 1 mM cysteine, 1 mM pyruvate, 100 units/ml penicillin, and 100 *μ*g/ml streptomycin. The tissue was cultured for 24 h at 37°C and 5% CO_2_ and 100%humidity ± 10^−4^ − 10 *μ*M of the ER stress inductor Thapsigargin (Santa Cruz Biotechnology, Inc., Dallas, Texas, USA) and 10-50 mM of 4-PBA (Santa Cruz Biotechnology, Inc., Dallas, Texas, USA).

### 2.3. Culture of C28/I2 Cells and Generation of ERp57 Knockout C28/I2 Cells

C28/I2 cells [23] were kept in DMEM (Biochrom, Berlin, Germany) supplemented with 10% FCS, 1% sodium pyruvate, 100 units/ml penicillin, and 100 *μ*g/ml streptomycin (complete DMEM) at 37°C, 5% CO_2_, and 100% humidity. To generate ERp57 knockout C28/I2 cells (C28/I2 KO cells), a cotransfection with an ERp57 CRISPR/Cas9 KO plasmid with a Green Fluorescent Protein (GFP) gene and an ERp57 homology directed repair (HDR) plasmid with puromycin resistance and Red Fluorescent Protein (RFP) genes (both from Santa Cruz Biotechnology, Inc., Dallas, Texas, USA) was performed according to the protocol of the manufacturer.

### 2.4. C28/I2 Micromass Cultures

Following counting, 20 *μ*l droplets of complete DMEM containing 4 × 10^5^ C28/I2 cells were pipetted into wells of a 24-well plate and left untouched for 3 h at 37°C, 5% CO_2_, and 100% humidity. Subsequently, 1 ml of complete DMEM was added. Micromass cultures were cultivated for 2 days before the medium was changed against serum-free DMEM supplemented with 60 *μ*g/ml *β*-aminopropionitrile fumarate, 25 *μ*g/ml sodium ascorbate, 1 mM cysteine, 1 mM pyruvate, 100 units/ml penicillin, and 100 *μ*g/ml streptomycin ± 10^−4^ − 10 *μ*M of Thapsigargin and/or 10-50 mM 4-PBA.

### 2.5. Isolation of RNA and Protein

Explant cultures were homogenized in 2 ml tubes with 2.8 mm beads in 750 *μ*l TRIzol (Life Technologies, Darmstadt, Germany) in a Bead Ruptor 12 homogenizer (Omni International Inc., NW Kennesaw, Georgia, USA) for 30 sec at 7.3 m/s. The TRIzol-cartilage suspension was transferred into a new 1.5 ml tube. Micromasses were directly suspended in 750 *μ*l TRIzol. RNA and protein isolation was performed according to the Invitrogen TRIzol reagent user guide. After drying, the isolated RNA pellet was resuspended in 25 *μ*l DEPC-H_2_O. The protein pellet was resuspended in 40 *μ*l chondroitinase buffer, containing 50 mM Tris/HCl, pH 8.0, 60 mM sodium acetate, 0.02% bovine serum albumin, and 1 mU chondroitinase ABC (Sigma-Aldrich, Taufkirchen, Germany). Digestion was performed for 1 h at 37°C, and 40 *μ*l 2-fold SDS-sample buffer was added before boiling for 5 min at 95°C.

### 2.6. DNase Digestion, cDNA Synthesis, and Semiquantitative RT-PCR

To eliminate residual DNA from RNA samples, the Ambion™ TURBO DNA-Free Kit (Thermo Fisher Scientific, Schwerte, Germany) was used according to the protocol of the manufacturer. cDNA synthesis was performed starting with 200 ng RNA with the RevertAid First Strand cDNA Synthesis Kit (Thermo Fisher Scientific, Schwerte, Germany) according to the protocol of the supplier. For the following PCR reactions, 1 *μ*l cDNA was mixed with 2.5 *μ*l 10-fold PCR buffer, 1.25 *μ*l 25 mM MgCl_2_, 1 *μ*l dNTPs (10 mM each), 1 *μ*l of each primer (10 pM/*μ*l, Biolegio, Nijmegen, Netherlands), and 1 *μ*l Taq polymerase (1 U/ml) and supplemented with DEPC-H_2_O to a final volume of 25 *μ*l. Cycling conditions were specific for different primers (see Table 1). For all samples, an initial denaturation step was performed for 10 min at 95°C, followed by 25-30 cycles at 95°C, 50-58°C, and 72°C (30 s each); a final extension was performed at 72°C for 10 min.

### 2.7. Immunoblot Analysis

Isolated proteins and prestained protein standards (Bio-Rad, Munich, Germany) were subjected to 4.5-15% SDS-polyacrylamide gel electrophoresis under reducing conditions. Proteins were electrotransferred onto nitrocellulose membranes (Schleicher & Schuell, Dassel, Germany) for 30 min at 25 V and ≤1 A with the Trans-Blot Turbo Blotting System (Bio-Rad, Munich, Germany). Membranes were blocked for 30 min in blocking buffer (5% dry skim milk+1% BSA in TBS containing 0.1% Tween 20 (TBS-T)), then incubated overnight with primary antibodies (see Table 2), washed with TBS-T, and finally incubated at room temperature for 1 h with peroxidase-coupled anti-mouse IgG antibody from donkey (Dianova, Hamburg, Germany, dilution 1 : 1,000) or peroxidase-coupled anti-rabbit IgG antibody from donkey (Amersham Bioscience, Freiburg, Germany, dilution 1 : 10,000). Signals were visualized with Super Signal® West Femto Maximum Sensitive Substrate (Thermo Fisher Scientific, Schwerte, Germany) following the procedure recommended by the supplier and detected with the chemiluminescence detector Fusion-SL4.2 MP (Peqlab Biotechnology, Erlangen, Germany).

### 2.8. Transmission Electron Microscopic (TEM) Analysis

Micromasses were fixed in 2% (*v*/*v*) formaldehyde and 2.5% (*v*/*v*) glutaraldehyde in 100 mM cacodylate buffer, pH 7.4, at 4°C overnight. After washing in PBS, samples were postfixed in 0.5% (*v*/*v*) osmium tetroxide and 1% (*w*/*v*) potassium hexacyanoferrate (III) in 0.1 M cacodylate buffer for 2 h at 4°C followed by washing with distilled water. After dehydration in an ascending ethanol series, specimens were incubated in propylene oxide for 2 × 15 min and embedded in Epon. Ultrathin sections were cut with an ultramicrotome, collected on copper grids, and negatively stained with 2% uranyl acetate for 10 min. Electron micrographs were taken at 60 kV with a Phillips EM-410 electron microscope using imaging plates (Ditabis, Pforzheim, Germany).

### 2.9. Statistical Analysis

Data are presented as means ± SD. Parametric (Student *t*-test) tests were performed using GraphPad Prism, V.6.0h (GraphPad Software Inc., San Diego, USA), with *p* < 0.05 determining the primary level of significance.

## 3. Results

### 3.1. Knockout of ERp57 Induces ER Stress in Chondrocytes *In Vitro*

**Figure 1:**
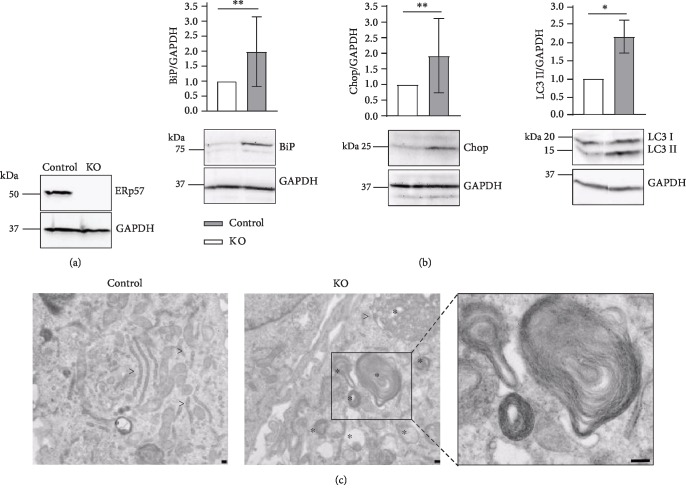
CRISPR/Cas9-induced knockout of ERp57 in C28/I2 cells results in ER stress and UPR signaling with subsequent induction of autophagy and apoptosis. (a) Immunoblot analysis of ERp57 in transfected and control C28/I2 cells with GAPDH as an internal control. ERp57 is entirely absent in C28/I2 cells after CRISPR/Cas9-induced knockout of ERp57 and puromycin selection of the transfected cells. Accordingly, the transfected cells are referred to as KO cells. (b) Immunoblot analysis of ER stress and autophagy marker proteins with GAPDH as an internal control. Compared to control cells set as 1, the investigated marker proteins BiP, Chop, and LC3 II are increased in ERp57 KO cells relative to the GAPDH levels. This demonstrates that the loss of ERp57 activity in the ER leads to a reduced protein folding capacity. Accumulating proteins in the ER induce ER stress with subsequent UPR signaling and induction of autophagy and apoptosis. *n* ≥ 3, ^∗^*p* < 0.05, and ^∗∗^*p* < 0.01. (c) Transmission electron microscopic analysis of ERp57 KO and control cells. Nontransfected C28/I2 cells (control) reveal regular stacks of rough ER (>) and low amounts of tiny autophagic vesicles (^∗^) in the cytoplasm. In contrast, ERp57 KO cells show large autophagic structures including bulk cytoplasm and entire organelles. Particularly striking are the autophagosomes including ER whorls (presented enlarged in the right panel). All bars = 200 nm.

To induce ER stress in cultured chondrocytes, an ERp57-deficient cell line was generated using the CRISPR/Cas9 knockout technology in C28/I2 cells. Subsequent to transfection and puromycin selection, the protein expression of ERp57 was analyzed by immunoblotting. The expression of glyceraldehyde 3-phosphate dehydrogenase (GAPDH) was tested in parallel as a loading control. As shown in Figure 1(a), nontransfected control cells showed a band above 50 kDa representing ERp57. The transfected C28/I2 cells, however, completely lacked this signal, indicating a successful KO strategy leading to a complete loss of ERp57. These cells, therefore, are referred to as ERp57 KO cells.

To characterize whether the loss of ERp57 results in ER stress, the protein expression of BiP, as the most important ER stress marker, was examined.  Figure 1(b) demonstrates that the loss of ERp57 in C28/I2 cells indeed resulted in enhanced UPR signaling via BiP. Relative to the GAPDH level, the intensity of the BiP signal on immunoblots increased approximately by 100%. In addition, Chop, a regulator of ER-stress-induced apoptosis, is augmented in the absence of ERp57. The increased BiP and Chop levels in ERp57 KO cells are highly comparable to the *in vivo* situation in cartilage-specific ERp57 KO mice investigated earlier [15].

Before UPR signaling induces apoptosis, autophagy is usually initiated as the last attempt to resolve the stress condition. By immunoblotting, an increase in microtubule-associated protein light chain 3 II (LC3 II), a protein widely used to monitor autophagy, was confirmed in ERp57 KO cells. The LC3 II/GAPDH ratio was found to be augmented by more than 100% in ERp57 KO cells compared to control cells.

In ERp57 KO cells, a larger number of autophagic vesicles was visible by TEM as demonstrated in Figure 1(c). Analysis of ultrathin sections of control cells revealed normal rough ER consisting of flattened cisternae, arranged in parallel. Such structures were extremely rare in ERp57 KO cells. Conversely, large amounts of autophagic vesicles and engulfment of ER whorls by vacuolar membranes occurred in ERp57 KO cells. In control cells, small autophagic vesicles were rarely detectable.

In summary, ERp57 KO C28/I2 cells reveal signs of ER stress comparable to chondrocytes in cartilage-specific ERp57 KO mice. Due to ER stress, UPR signaling is induced and autophagy as well as apoptosis is initiated. Therefore, the generated ERp57 KO cells are suitable for testing of ER-stress-reducing drugs.

### 3.2. Thapsigargin Is a Potent ER Stress Inductor in C28/I2 Cells

**Figure 2:**
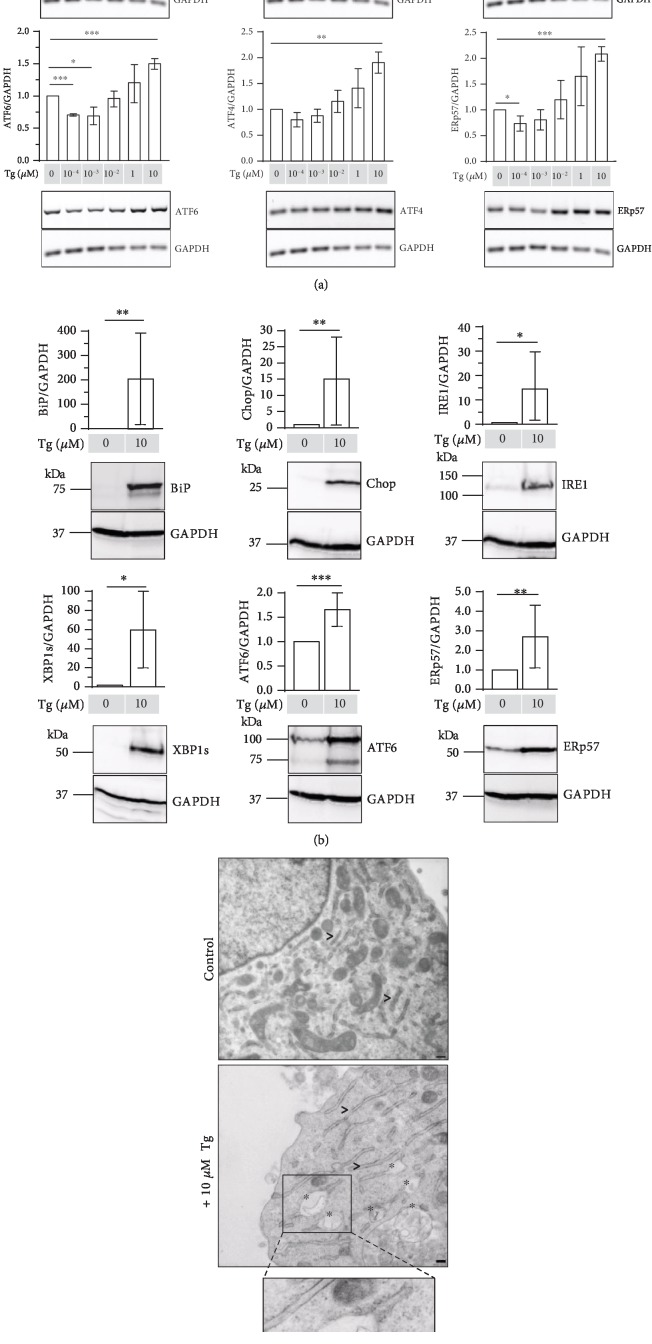
Thapsigargin is a potent inductor of ER stress in C28/I2 cells. (a) RT-PCR analysis of C28/I2 micromass cultures stimulated with rising concentrations of the ER stress inductor Thapsigargin (Tg). mRNA expression levels of BiP, Chop, XBP1 spliced (XBP1s), ATF6, ATF4, and ERp57 relative to GAPDH are shown with mRNA levels of unstimulated cells set as 1. The PCR results demonstrate a dose-dependent increase of both ER stress markers with increasing Tg concentrations. *n* = 3, ^∗^*p* < 0.05, ^∗∗^*p* < 0.01, and ^∗∗∗^*p* < 0.001. (b) Immunoblot analysis of C28/I2 micromass cultures stimulated with 10 *μ*M of the ER stress inductor Thapsigargin (Tg). Relative to GAPDH as internal control, all investigated marker proteins BiP, Chop, IRE1, XBP1s, ATF6, and ERp57 are significantly increased in C28/I2 cells in the presence of 10 *μ*M Tg compared to nonstimulated control cells set as 1. *n* ≥ 3, ^∗^*p* < 0.05, ^∗∗^*p* < 0.01, and ^∗∗∗^*p* < 0.001. (c) Transmission electron microscopic analysis of C28/I2 control cells cultured in micromasses in the presence or absence of 10 *μ*M Tg. C28/I2 cells w/o Tg (control) reveal regular stacks of rough ER (>). Thapsigargin stimulation of C28/I2 cells leads to distended ER structures (^∗^) suggesting an accumulation of un- or misfolded proteins in the ER. All bars = 200 nm.

C28/I2 cells were also treated with Thapsigargin (Tg), a specific inhibitor of the ER Ca^2+^-ATPase decreasing ER calcium levels. The calcium-dependent chaperones calnexin and calreticulin and in consequence ERp57, lose their activity, leading to ER stress [24]. Results of mRNA expression analysis in Figure 2(a) demonstrate that Tg dose-dependently increased the mRNA expression of BiP and Chop. Moreover, the mRNA of XBP1s (activated by IRE1), ATF6 (initiating the ATF6 pathway), and ATF4 (activated by PERK) were increased, suggesting that all three UPR signaling pathways are triggered by Tg. To show that the expression of downstream target genes also changes, the expression of ERp57 was checked. The ERp57/GAPDH mRNA ratio significantly increased at a concentration of 10 *μ*M Tg. Therefore, 10 *μ*M Tg was used in further experiments.

In addition to mRNA analyses, protein levels were examined by immunoblotting. As presented in Figure 2(b), all investigated ER stress marker proteins BiP, Chop, IRE1, XBP1s, and ATF6 and the target protein ERp57 were significantly increased in the presence of 10 *μ*M Tg. These results confirm that Tg is a highly potent ER stress inductor activating the UPR through all three existing ER stress sensor proteins.

Figure 2(c) shows the results of TEM analysis. Stimulation of C28/I2 micromass cultures with 10 *μ*M Tg led to distended ER structures suggesting an accumulation of misfolded proteins in the ER and ER stress. Similar dilated ER cisternae were earlier detected in chondrocytes of cartilage-specific ERp57 KO mice [15].

Taken together, Tg is applicable to chemically induce various intensities of ER stress in C28/I2 cells. This makes Tg suitable to test ER-stress-mitigating drugs in cell culture.

### 3.3. 4-PBA Efficiently Reduces ER Stress *In Vitro*

**Figure 3:**
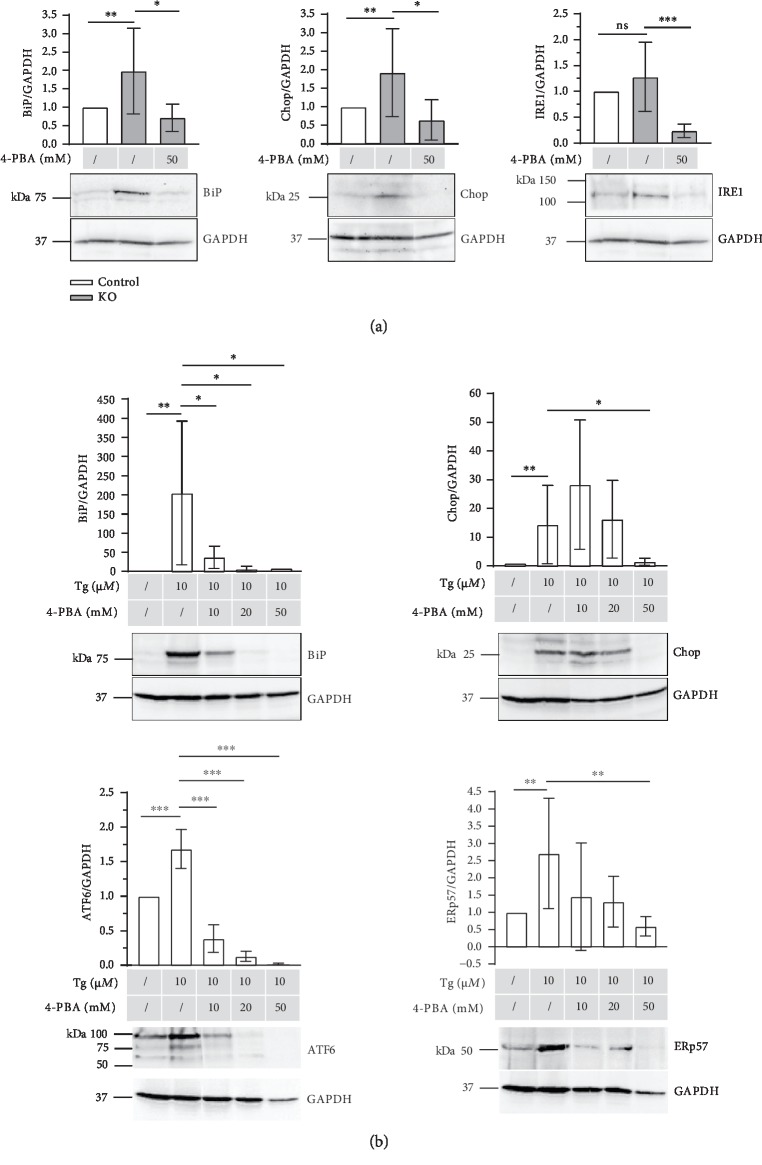
4-PBA efficiently reduces various levels of ER stress *in vitro*. (a) Immunoblot analysis of micromass cultures of ERp57 KO and control cells to analyze ER marker proteins in the presence of 50 mM 4-PBA. Unstimulated C28/I2 cells served as control and their protein/GAPDH ratios were set at 1. As expected, BiP, Chop, and IRE1 are increased in ERp57 KO cells compared to the control cells in the absence of 4-PBA. The addition of 50 mM 4-PBA to ERp57 KO cells reduces ER stress marker protein expression levels to or even below control levels. *n* ≥ 6, ^∗^*p* < 0.05, ^∗∗^*p* < 0.01, and ^∗∗∗^*p* < 0.001. (b) Immunoblot analysis of micromass cultures of Thapsigargin- (Tg-) stimulated C28/I2 cells to analyze ER marker proteins in the presence of different concentrations of 4-PBA. Unstimulated C28/I2 cells served as control and their protein/GAPDH ratios were set as 1. As expected, BiP, Chop, ATF6, and ERp57 are increased in the presence of 10 *μ*M Thapsigargin compared to the unstimulated control cells. The addition of 10, 20, and 50 mM 4-PBA to micromass cultures of Thapsigargin-stimulated C28/I2 cells dose-dependently reduced ER stress marker protein expression levels even under the control levels. *n* ≥ 3, ^∗^*p* < 0.05, ^∗∗^*p* < 0.01, and ^∗∗∗^*p* < 0.001.

Next, the effects of 4-Phenylbutyric acid (4-PBA), which acts as a chemical chaperone attenuating ER stress and UPR signaling in different cell types, was tested in both *in vitro* ER stress models mentioned above (Figure 3).

The effects of 50 mM 4-PBA were examined on the protein level of micromasses of ERp57 KO cells first. Data presented in Figure 3(a) reveal that the levels of the ER stress marker proteins BiP, Chop, and IRE1 were increased in ERp57 KO cells relative to the control cells, indicating higher ER stress levels in the absence of ERp57. The addition of 50 mM 4-PBA to ERp57 KO cells was effective and reduced the protein levels of all three ER stress markers. In case of IRE1, the ER stress protein was reduced even below the control cell level. This is in contrast to BiP, which is due to its multifunctional role, expressed at relatively high constitutive levels even without ER stress.

Secondly, 10, 20, and 50 mM 4-PBA were added to C28/I2 micromass cultures treated with 10 mM Tg. The upregulation of BiP, Chop, ATF6, and ERp57 in the presence of 10 *μ*M Tg was demonstrated above.  Figure 3(b) illustrates that the addition of 4-PBA dose-dependently reduced all ER stress marker proteins suggesting a potent ER-stress-relieving function of 4-PBA. The ATF6/GAPDH levels were reduced below the control levels of unstimulated C28/I2 cells with 20 and 50 mM 4-PBA. On the ATF6 blots, not only the full length but also cleaved, active forms of the protein are detectable. In the case of Chop, 50 mM 4-PBA is required for a reduction to control levels. It is worthwhile to mention that the Chop signals tend to be slightly higher in the presence of 10 mM 4 − PBA + 10 mM Tg than in the presence of 10 mM Tg alone. Chop runs as a doublet, which may reflect the phosphorylation of the protein that enhances its ability to function as a transcriptional activator. For quantification, all ATF6 and Chop bands were selected.

Taken together, these data demonstrate that 4-PBA efficiently eliminates ER stress in cell cultures, regardless of whether ER stress is induced by ERp57 knockout or Tg.

### 3.4. 4-PBA Efficiently Diffuses into Cartilage and Alleviates Tg-Induced ER Stress

**Figure 4:**
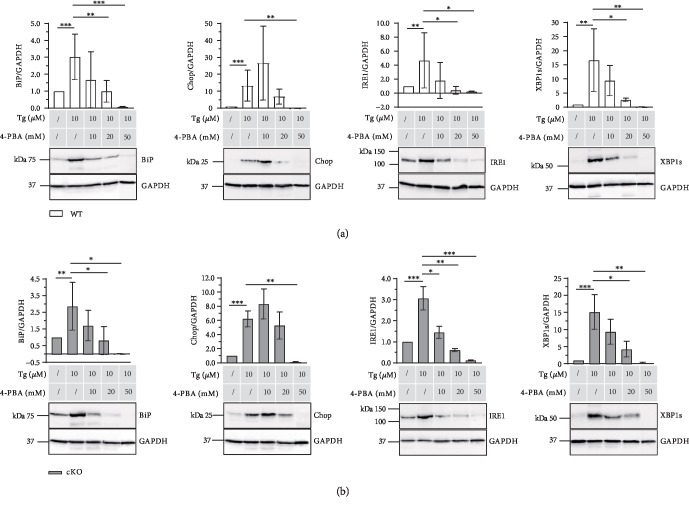
4-PBA relieves ER stress in explant cultures of mouse hip and knee cartilage. (a) Immunoblot analysis of knee and hip cartilage isolated from WT mice and cultured *ex vivo* in explant cultures. ER marker proteins were analyzed in the presence of 10 *μ*M Tg and different concentrations of 4-PBA. Unstimulated cartilage samples served as control and their protein/GAPDH ratios were set at 1. BiP, Chop, IRE1, and XBP1s protein levels increased in the presence of 10 *μ*M Thapsigargin compared to the unstimulated control. The addition of 10, 20, and 50 mM 4-PBA to the Thapsigargin-stimulated explant cultures dose-dependently reduced the ER stress marker protein expression levels back to the control levels. *n* ≥ 4, ^∗^*p* < 0.05, ^∗∗^*p* < 0.01, and ^∗∗∗^*p* < 0.001. (b) Immunoblot analysis of knee and hip cartilage isolated from cartilage-specific ERp57 KO mice (ERp57 cKO) and cultured *ex vivo* in explant cultures. ER marker proteins were analyzed in the presence of 10 *μ*M Tg and different concentrations of 4-PBA. Unstimulated cartilage samples served as a control and their protein/GAPDH ratios were set at 1. Corresponding to WT cartilage, BiP, Chop, IRE1, and XBP1s protein levels increased in ERp57 KO cartilage in the presence of 10 *μ*M Thapsigargin compared to the unstimulated control. Likewise, the addition of 10, 20, and 50 mM 4-PBA to the Thapsigargin-stimulated explant cultures dose-dependently reduced the ER stress marker protein expression levels back to the control levels. *n* ≥ 3, ^∗^*p* < 0.05, ^∗∗^*p* < 0.01, and ^∗∗∗^*p* < 0.001.

To analyze if 4-PBA is applicable in cartilage tissues, hip and knee cartilage explant cultures of ERp57 cKO and WT animals were examined.  Figure 4 reveals that 10 *μ*M Tg significantly increased the protein levels of the ER stress marker proteins BiP, Chop, IRE1, and XBP1s in WT (Figure 4(a)) and cKO (Figure 4(b)) chondrocytes in knee and hip cartilage cultured *ex vivo*. This suggests that the data obtained in micromass cultures of C28/I2 cells accordingly apply to chondrocytes within cartilage.

The same is true for 4-PBA. 4-PBA efficiently reduced the Tg-stimulated ER stress in explant cultures. Comparable to the micromass cultures of C28/I2 cells, 4-PBA dose dependently reduced the protein levels of BiP and other ER stress marker proteins such as IRE1 and XBP1s. The addition of 20 mM 4-PBA lowered the ER stress marker protein to the control levels without Tg.

In both WT and cKO cartilage explant cultures, the immunoblots of Chop are different. Analogous to the micromass cultures, the addition of 10 mM 4-PBA slightly amplified the Chop signals compared to the values obtained with only 10 *μ*M Tg. Furthermore, the addition of 20 mM 4-PBA is not sufficient but the application of 50 mM 4-PBA lowers the ER stress to control levels without Tg.

These data suggest that 4-PBA (1) is capable of diffusing into the cartilage tissues, (2) is able to reach the target structures in the ER, and (3) with high probability, is applicable as an ER-stress-reducing drug for the treatment of ER-stress-related cartilage diseases.

## 4. Discussion

So far, pharmacological intervention for the treatment of ER-stress-induced cartilage diseases is not available. Therefore, it is of great interest to understand ER stress and UPR signaling in chondrocytes in detail. The aim of this study was to evaluate the effects of 4-Phenylbutyric acid (4-PBA) to relieve ER stress in chondrocytes. As reviewed by Kolb et al. [22], 4-PBA is ascribed many effects in various cellular systems and concentrations. 4-PBA is currently used as an ammonia scavenger to treat urea cycle disorders [25] and is also thought to act as a weak histone deacetylase inhibitor [26]. However, 4-PBA is mostly applied as a low molecular weight chemical chaperone to improve protein folding in ER-stress-related pathologies such as diabetes [27], cystic fibrosis [28], sickle cell disease [29], and neurodegenerative diseases [30].

In this study, the ER-stress-resolving function of 4-PBA was confirmed for chondrocytes. We demonstrated that 4-PBA effectively reduces ER stress and the subsequent UPR signaling as well as apoptosis. Moreover, 4-PBA efficiently diffuses into cartilage and reaches the ER of chondrocytes as the target structure in explant cultures. A sufficient transport of therapeutic agents into cartilage is difficult because of the highly bradytrophic, dense, and avascular nature of this tissue [31]. However, 4-PBA is a small phenyl-substituted fatty acid and, therefore, was assumed to penetrate cartilage quickly. The presence as a negatively charged ion in solution also does not seem to interfere with diffusion [22]. For a future treatment of cartilage diseases, oral intake appears feasible since it has been shown in mice that the feeding of 4-PBA was sufficient to achieve appropriate concentrations at least in blood plasma [32].

Moreover, we discovered that 4-PBA resolves ER stress in chondrocytes under different conditions. We presented efficacy in micromasses of ERp57 KO cells in explant cultures of cartilage obtained from ERp57 cKO mice and in Thapsigargin-stimulated C28/I2 cells. It is relevant to know that ER stress in cartilage is essential under physiological and pathological conditions. Physiologically, ER stress occurs during bone development when high amounts of proteins are synthesized under hypoxic conditions and at low energy levels [33, 34]. In addition, ER stress is triggered by pathological conditions, such as Ca^2+^-ion imbalances [35], expression of mutant proteins leading to aggregation in the ER [13], deficiencies in ER chaperones [36], mutations in UPR signaling factors [37, 38], or deficiencies in the degradation of aggregated proteins [39, 40]. In any case, ER stress substantially impairs protein synthesis, chondrocyte proliferation, and/or differentiation or regulation of autophagy and apoptosis. As a result, cartilage diseases arise no matter if single or multiple events are affected. With this knowledge in mind, the following should be considered: (1) During therapeutic intervention, only excessive, harmful forms of ER stress should be diminished. A total elimination of ER stress could result in a dysregulation of essential metabolic pathways in chondrocytes. (2) Since ER stress is triggered by different forms of ER imbalance, the induced pathologies each require individual pharmacological treatment. Thus, it is very likely that the aggregation of proteins in the ER occurring due to mutations in ECM proteins cannot be cured by the same means in any case. If a given mutation inevitably leads to a misfolding of a protein, an additional chaperone, either artificially supplied or naturally expressed in higher concentrations after UPR signaling, may fail. This might explain why 4-PBA treatment was ineffective in a mouse model of MED with the Matrillin-3 mutation p.V194D [32] and in a mouse model of MCDS with a p.Asn617Lys mutation of collagen type X [41]. However, this does not imply that the application of chemical chaperones is inefficient in cartilage diseases in general.

We established that 4-PBA reduced ER stress in chondrocytes missing ERp57. Here, an absent chaperone was replaced by a chemical one. ERp57 is critically involved in protein folding processes, especially in the disulfide bridge formation in glycoproteins with unstructured disulfide-rich domains [7–10]. This function was demonstrated to be essential during development, as the constitutive ERp57 KO mice show embryonic lethality at E13.5 [42, 43]. To analyze the role of ERp57 in chondrocytes, we generated a cartilage-specific ERp57 KO mouse. This mouse model revealed that the deficiency of ERp57 in chondrocytes is sufficient to induce ER stress with subsequent UPR signaling and to cause an ER-stress-related bone phenotype resembling a chondrodysplasia [15]. Here, we demonstrated, that 4-PBA reduces ER stress in *ex vivo* cultures of knee and hip cartilage of these mice. However, whether 4-PBA treatment indeed rescues the chondrodysplasia-like bone phenotype of ERp57 cKO mice is to be analyzed in the future. 4-PBA, however, was not only shown to be effective in our experiments, but also in an experimental rat OA model. Tang et al. demonstrated that 4-PBA inhibited the ER-stress-induced apoptosis of articular chondrocytes and thereby exhibited a protective effect against cartilage degradation during OA [44]. Tauroursodeoxycholic acid seems to have comparable effects. This drug was applied in cell cultures of human chondrocytes isolated from OA patients and reduced the Tunicamycin-induced expression of the ER stress marker proteins BiP and CHOP as well as apoptosis of the cultured cells [45]. ER stress in chondrocytes, whether or not related to chondrodysplasias or osteoarthritis, may be due to different defects. Therefore, various cellular processes associated with ER stress may be used as potential therapeutic targets in chondrocytes. One option is a selective modulation of single UPR signaling pathways. It was shown earlier that ER-stress-induced activation of PERK initiates the phosphorylation of the eukaryotic translation initiation factor 2 alpha. This shuts down the global protein synthesis but also induces the preferential translation of ATF4. ATF4 activates downstream an inappropriate expression of SOX9, reverting chondrocyte differentiation and generating the disease-causing developmental defects in MCDS [46]. By targeting the ER stress sensor PERK, the abovementioned UPR signaling changes and the disease-triggering chondrocyte differentiation defects should be prevented. Indeed, the application of a chemical inhibitor of PERK signaling ameliorated the chondrodysplasia phenotype in the MCDS mouse model 13del [47].

Therapies against ER stress may also include the enhancement of ER-associated degradation or autophagy and the inhibition of ER-stress-induced inflammation [48]. One possible candidate to stimulate autophagy is trehalose. This drug was actually shown to ameliorate OA development in a destabilized medial meniscus mouse OA model by the inhibition of ER-stress-induced apoptosis especially during late stage OA [49]. Carbamazepine, another known autophagy stimulator, additionally stimulates degradation via proteasomes [50]. In a MCDS mouse model, the application of Carbamazepine led to a significant improvement of the bone phenotype [50]. This originates from a stimulated proteolysis of misfolded collagen type X triggering the disease. However, carbamazepine did rescue the phenotype only partially. Therefore, the application of different ER-stress-reducing drugs in combination seems appropriate. Suppression of apoptosis could be a useful therapeutic option, too [14]. Guo et al. demonstrated that the overexpression of XBP1s in cartilage explants from human OA patients blocked ER-stress-induced chondrocyte cell death [51].

In conclusion, ER stress is an important player in cartilage diseases. It induces various forms of chondrodysplasias and is a pathogenic factor in osteoarthritis. Accordingly, ER stress signaling pathways provide selective targets for potential therapeutic intervention. One therapeutic option is the application of the small molecular chemical chaperone 4-PBA, which efficiently diffuses into cartilage tissues, reaches its target within the ER of chondrocytes, and ameliorates UPR signaling. Other promising drugs that might be used in parallel are inhibitors of single UPR signaling pathways, activators of ER-associated degradation, and autophagy or inhibitors of excessive apoptosis. However, further *in vivo* studies are needed to assess whether 4-PBA alone or in combination is applicable to achieve improvement in ER-stress-related cartilage disorders in patients.

## Figures and Tables

**Table 1:** PCR primer sequences and PCR conditions.

Primer	Sequence (5′>3′)	Annealing temperature	Number of cycles
ERp57 forward ERp57 reverse	AGAGACTTGCACCTGAGTAT GCAGTCCTAGGTCCATCATA	52°C	28
XBP1s forward XBP1s reverse	GAGTCCGCAGCAGGTG CCGCCAGAATCCATGGGG	52°C	30
GAPDH forward GAPDH reverse	GAAGGTGAAGGTCGGAGTCAAC CAGAGTTAAAAGCAGCCCTGGT	55°C	25
BiP forward BiP reverse	GGGGACCACCTACTCCTGCGTC ATAGGACGGCGTGATGCGGT	58°C	25
Chop forward Chop reverse	TGTTAAAGATGAGCGGGTGGCA TGCTTTGGTGCTGCTTTCAGGTGT	58°C	25
ATF6 forward ATF6 reverse	TTATCAGTTTACAACCTGCA CTAGGGACTTTAAGCCTCTG	50°C	30

**Table 2:** Primary antibodies for immunoblot analysis.

Antibody	Supplier	Dilution
ERp57, rabbit pAB	Enzo Life Sciences, Lörrach, Germany	1 : 1000
BiP, rabbit mAB	Cell Signaling Technology, Frankfurt, Germany	1 : 1000
Chop, mouse mAB	Cell Signaling Technology, Frankfurt, Germany	1 : 1000
IRE1, mouse mAB	Santa Cruz Biotechnology, Inc., Dallas, Texas, USA	1 : 200
XBP1s, rabbit pAB	BioLegend, Nijmegen, Netherlands	1 : 500
ATF6, rabbit pAB	Santa Cruz Biotechnology, Inc., Dallas, Texas, USA	1 : 200
GAPDH, rabbit mAB	Cell Signaling Technology, Frankfurt, Germany	1 : 1000

## Data Availability

All data used to support the findings of this study are included within the article or are available from the corresponding author upon request.
